# Cellular automaton-based model for radiation-induced bystander effects

**DOI:** 10.1186/s12918-015-0235-2

**Published:** 2015-12-07

**Authors:** Yuya Hattori, Akinari Yokoya, Ritsuko Watanabe

**Affiliations:** Research Group for Radiation Effect Analysis, Japan Atomic Energy Agency, 2-4, Shirakata Shirane, Tokai, Ibaraki, 319-1195 Japan; Research Group for Radiation and Biomolecular Science, Japan Atomic Energy Agency, Ibaraki, 319-1195 Japan

**Keywords:** Cell cycle, DNA double strand break, Intercellular signaling, Modeling, Radiation-induced bystander effect

## Abstract

**Background:**

The radiation-induced bystander effect is a biological response observed in non-irradiated cells surrounding an irradiated cell. The bystander effect is known to be induced by two intercellular signaling pathways, the medium-mediated pathway (MDP) and the gap junctional pathway (GJP). To investigate the relative contribution of each signaling pathway, we have developed a mathematical model of the cellular response through these two pathways, with a particular focus on cell-cycle modification.

**Methods:**

The model is based on a cellular automaton and consists of four components: (1) irradiation, (2) generation and diffusion of intercellular signals, (3) induction of DNA double-strand breaks (DSBs), and (4) cell-cycle modification or cell death. The intercellular signals are generated in and released from irradiated cells. The signals through the MDP and the GJP are modeled independently based on diffusion equations. The irradiation and both signals raise the number of DSBs, which determines transitions of cellular states, such as cell-cycle arrest or cell death.

**Results:**

Our model reproduced fairly well previously reported experimental data on the number of DSBs and cell survival curves. We examined how radiation dose and intercellular signaling dynamically affect the cell cycle. The analysis of model dynamics for the bystander cells revealed that the number of arrested cells did not increase linearly with dose. Arrested cells were more efficiently accumulated by the GJP than by the MDP.

**Conclusions:**

We present here a mathematical model that integrates various bystander responses, such as MDP and GJP signaling, DSB induction, cell-cycle arrest, and cell death. Because it simulates spatial and temporal conditions of irradiation and cellular characteristics, our model will be a powerful tool to predict dynamical radiobiological responses of a cellular population in which irradiated and non-irradiated cells co-exist.

**Electronic supplementary material:**

The online version of this article (doi:10.1186/s12918-015-0235-2) contains supplementary material, which is available to authorized users.

## Background

It has long been desired to understand low-dose effects of ionizing radiation on living systems. From a practical viewpoint, the risks of low-dose radiation have been discussed for clinical diagnosis and therapy, as well as for radiation control around a nuclear disaster site. Even though only a limited number of cells in the population are exposed to doses of radiation below 1 Gy, it has been widely recognized that radiobiological effects, such as chromosome aberrations [1] and micronucleation or apoptosis [2], can be induced not only in the irradiated cells but also in non-irradiated cells. The transfer of radiation effects from irradiated to non-irradiated cells is termed the “bystander effect”. Bystander effects are activated by two intercellular signaling pathways: the medium-mediated pathway [3] and the gap junctional pathway [4]. To understand the mechanisms of various bystander responses, the detailed dynamics of these two pathways is needed.

One powerful approach to understand the mechanism of bystander signal transfer is modeling the processes mathematically. In the last decade, several studies have reported models of the bystander effect. A Bystander and Direct (BaD) model, based on surviving fractions, was proposed by Brenner et al. [5]. In the BaD model, cell death could be induced both by direct exposure to ionizing radiation and by bystander signals. Ebert et al. developed a surviving fraction model taking into account the density of bystander signals and the probabilities of interaction between a cell and the bystander signals [6]. Several surviving fraction models or simulations have been reported that focus on the spatial and temporal kinetics of the bystander effect through the medium-mediated pathway [7, 8]. These models simulate the cellular population response as a surviving fraction and allow easy analysis of experimental data. However, it is very difficult to simulate the individual kinetics of cellular responses using these previous models. Khvostunov and Nikjoo developed a biophysical model that takes into account the diffusion of bystander signals and individual cell death (bystander diffusion modeling: BSDM) [9, 10]. In this model, the reactions of signals were calculated using the Monte Carlo method. Several similar attempts have also been made [11–13]. Richard et al. developed a simple deterministic model based on a cellular automaton [14, 15], which a useful model to describe the dynamics of a population of proliferating cells. Another model includes the repair of cell damage and cell death for both tumor cells and normal cells [16]. Although these models could simulate individual cellular responses caused by signaling through the medium-mediated pathway, the gap junctional pathway has not been fully taken into account.

In some of these studies [5, 6, 9, 10, 12–15], biological end-point of bystander cells were simply described as binary states, namely, “life” or “death”. However, this is not enough to represent the complex responses of bystander cells, which could have various intermediate states between life and death, such as arrest or elongation of the cell cycle or the G0 state. It has also been widely recognized that cell-cycle arrest would be strongly dependent on the number of double-strand breaks (DSBs) in an irradiated cell, although there have been very few reports on bystander cell-cycle modifications [17].

We have previously developed a model framework for bystander effects on cell-cycle modification or cell death mediated by intercellular signaling through both the medium-mediated pathway and the gap junctional pathway [18]. This model could calculate the time course of the individual cellular responses, however, cellular DNA damage was not fully taken into account, even cell-cycle arrest is thought to be one of the primary processes to secure sufficient time for repairing DNA damage, such as DNA DSBs.

In this study, we improved our model to simulate the induction of DSBs, cell-cycle modification, and cell death arising in non-irradiated cells. To model the inhomogeneous diffusions through the gap junctional pathway, we introduced “diffusion-direction constants” that define the direction of signal diffusion by referring to the states of adjacent cells. Furthermore, we have also introduced a biological “apparatus”, a virtual clock regulating cell cycles by cell-cycle checkpoints which function based on the number of DSBs produced. These are major advantages of our bystander model over previous ones. Using this model, we have analyzed in detail the dynamics of populations of cells, particularly cell-cycle modification or cell death. Various conditions, such as radiation dose or the relative contribution of each signaling pathway were carefully examined.

## Methods

**Fig. 1:**
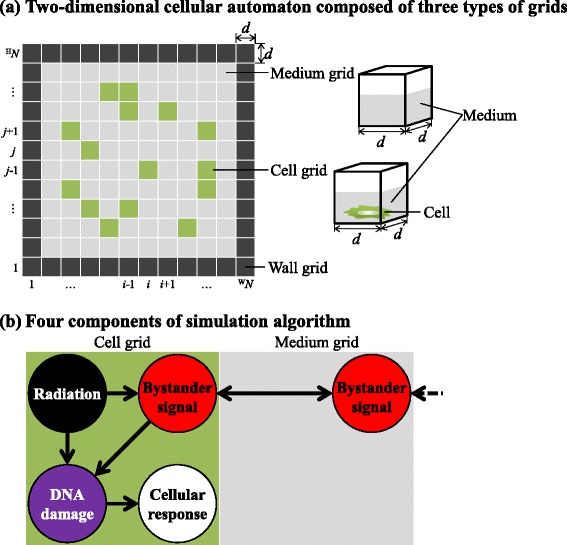
Schematic layout of the model of radiation-induced bystander-effects. **a** Two-dimensional cellular automaton composed of three types of grids : cell (*green*), medium (*gray*), and wall (*black*). **b** Four components of the simulation algorithm: (1) irradiation of the cells (*black*); (2) generation of bystander signals in the irradiated cells and diffusion of the signals to surrounding cells (*red*); (3) induction of cellular DNA damage induced by irradiation or bystander signals (*purple*); and (4) cellular response caused by DNA damage (*white*)

The simulation system is based on a two-dimensional cellular automaton, as shown in Fig. 1a. The grids are categorized into three types: cell, medium, and wall. Each cell grid consists of a cell and culture medium. Each medium grid is filled entirely with culture medium. The wall grids represent a cell-culture container.

The simulation algorithm consists of four components: (1) irradiation of the cells, (2) generation of bystander signals in the irradiated cells and diffusion of these signals to surrounding cells, (3) cellular DNA damage induced by irradiation or bystander signals, and (4) cellular responses to the DNA damage. Cell-cycle modification and cell death were simulated as cellular responses. These four components relate to each other as shown in Fig. 1b. The spatial and temporal evolution of the cellular population can be simulated by following the temporal progress of these components in individual cells.

### Irradiation to cells

The biological effectiveness of ionizing irradiation generally depends on both absorbed dose and dose rate. The dose is usually described as the average dose calculated by dividing the total absorbed energy by the mass of the target cellular population. However, the radiation dose is really the result of the random traversal of radiation tracks and the absorbed dose in each cell is non-uniform, particularly following low-dose irradiation.

We therefore assumed that a single radiation track passing through a cell gives a fixed radiation dose, *D*_1track_, which is set to correspond to an elementary dose. The number of radiation tracks in each cell is calculated based on random function, and the absorbed dose is calculated as the accumulation of the radiation tracks. The number of radiation tracks in grid position (*i*,*j*) at time *t* is represented by a random variable *K*_*i*,*j*_(*t*). Therefore, the absorbed dose, *R*_*i*,*j*_(*t*), in (*i*,*j*) at *t* is

(1) $$\begin{array}{@{}rcl@{}} R_{i,j}(t) = D_{\mathrm{1track}}K_{i,j}(t). \end{array} $$

The value of *K*_*i*,*j*_(*t*) is determined according to a Poisson distribution:

(2) $$\begin{array}{@{}rcl@{}} {\!\!~}^{\mathrm{K}}P_{i,j}\left({~\!\!}^{\mathrm{K}}n\right) = \frac{\left({~\!\!}^{\mathrm{K}}a\right)^{{~\!\!}^{\mathrm{K}}n}e^{-{~\!\!}^{\mathrm{K}}a}}{{~\!\!}^{\mathrm{K}}n!} \end{array} $$

where ^K^*P*_*i*,*j*_(^K^*n*) is the probability of ^K^*n* radiation tracks arising in grid (*i*,*j*) over time interval *Δt* and ^K^*a* is the average number of radiation tracks passing through a grid in interval *Δt*.

The values of *D*_1track_ and ^K^*a* can be determined for various radiation types. For example, when cells are irradiated by ^60^Co *γ*-rays, *D*_1track_=0.001 Gy. This value can be derived from the average dose given by secondary electrons generated by *γ*-irradiation at a very low dose rate [19]. In the case of heavier ions, such as helium or carbon ions, *D*_1track_ can be higher.

### Generation and diffusion of intercellular signaling

Intercellular signaling is described by the diffusion of transmitters into surrounding grids. We considered two kinds of radiation-induced signal: one that is transferred through the medium-mediated pathway (MDP) and another one through the gap junctional pathway (GJP). It has been mostly understood that some substances, such as NO radicals or cytokines, are major signals transferred through MDP [20, 21]. On the other hand, for the GJP, not all substances involved in the signaling have been identified. In general, cells communicate using messenger molecules such as c-AMP, calcium ions, inositol trisphosphate, ATP, or other nucleotides through gap-junctions [22–25] with a hexameric structure consisting of various connexin proteins [26]. Among these chemicals, the calcium ion is known to play a major signaling role in various biological processes. Thus, we assume that the virtual signal through the GJP has the same characteristics as that of calcium ions in our model.

The two virtual signals were assumed to have the following properties: (1) there are no interactions between the signal molecules; (2) the signals are proportional to absorbed dose and degraded exponentially over time; and (3) the signals are diffusion-controlled. Previously reported bystander-signal molecules, such as NO radicals or cytokines, are not likely to specifically recognize and interact with each other like proteins, although they could be degraded non-specifically by radical reactions or the digestive action of cells. Therefore, we made the no interaction and exponential decay assumptions. Assumption (2) seemingly contradicts the experimental result reported by Hu et al. that there was no dose dependence of the number of cells possessing DSBs [27]. In their study, bystander cells were counted as “DSB-positive cells” irrespective of the number of DSBs actually arising in the cell. Thus it is difficult to directly compare the number of bystander signals with the number of resulting DSBs. In order to avoid possibly underestimating the efficiency of the number of bystander signals or induced DSBs, assumption (2) is not unrealistic as a first order of approximation. Indeed, there has been further experimental evidence of the dose dependence of bystander cell death in the low-dose region [28]. In higher-dose regions, however, there have been no reports of a dependence of produced signals on dose. To validate our simulation results, experimental investigations should be performed in future (see the ‘Discussion and conclusions’).

The diffusion equation is generally discretized in the following way (see Additional file 1):

(3) $$\begin{array}{@{}rcl@{}} {\phi}_{i,j}(t+{\Delta}t) & = & {\phi}_{i,j}(t)+ \frac{{\Delta}t}{4d^{2}}{\!\!~}^{\mathrm{\phi}}{\!}w \left[2\sum_{k_{1},l_{1}}\left({\phi}_{k_{1},l_{1}}(t)-{\phi}_{i,j}(t)\right) \right. \\ &\quad &\left.+ \sum_{k_{2},l_{2}}\left({\phi}_{k_{2},l_{2}}(t)-{\phi}_{i,j}(t)\right){\vphantom{\frac{1}{2}}}\right] \end{array} $$

where *ϕ*_*i*,*j*_(*t*) is the signal concentration in grid (*i*,*j*), *Δt* is the time interval, *d* is the width of the grid, ^ϕ^*w* is the diffusion coefficient, and (*k*,*l*) represents the positions of the eight grids surrounding grid (*i*,*j*). Based on the distance of (*k*,*l*) from (*i*,*j*), we categorized the surrounding grids into two parts, indexed by (*k*_1_,*l*_1_) and (*k*_2_,*l*_2_). The former contains grids located to the right, left, above and below the central grid, and the latter contains the skew grids. That is, (*k*_1_,*l*_1_) is (*i*+1,*j*), (*i*−1,*j*), (*i*,*j*+1), or (*i*,*j*−1), while (*k*_2_,*l*_2_) is (*i*+1,*j*+1), (*i*−1,*j*+1), (*i*+1,*j*+1), or (*i*−1,*j*−1).

**Fig. 2:**
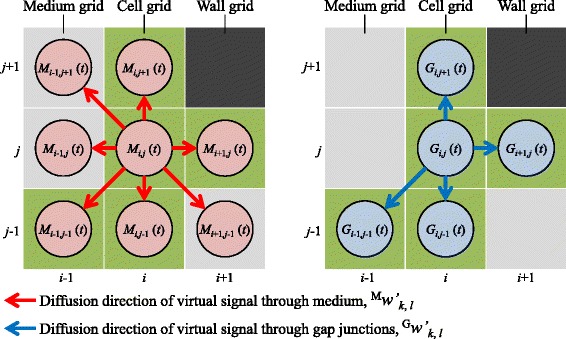
Diffusion directions of the virtual signals. The diffusion-direction constants indicate the directions of signal diffusion through the medium-mediated pathway (MDP) and the gap junctional pathway (GJP). The virtual signal through the MDP diffuses into surrounding cell and medium grids. The virtual signal through the GJP diffuses into only surrounding cell grids

Because the virtual signal diffuses into both cell grids and medium grids by the MDP, the signal is homogeneously transferred in all directions. On the other hand, the virtual signal through the GJP transferred to only nearby cell grids, resulting in an inhomogeneous diffusion of the signal (shown in Fig. 2). To control the direction of signal diffusion, we defined diffusion-direction constants, ^M^*wk*,*l*′ and ^G^*wk*,*l*′:

(4) $$\begin{array}{@{}rcl@{}} {\!\!~}^{\mathrm{M}}{\!}w'_{k,l} & = & \left\{ \begin{array}{cl} {\!\!~}^{\mathrm{M}}{\!}w & (\text{grid} (k,l): \text{cell grid}) \\ {\!\!~}^{\mathrm{M}}{\!}w & (\text{grid} (k,l): \text{medium grid}) \\ 0 & (\text{grid} (k,l): \text{wall grid}) \end{array} \right. \\  \end{array} $$

(5) $$\begin{array}{@{}rcl@{}} {\!\!~}^{\mathrm{G}}{\!}w'_{k,l} & = & \left\{ \begin{array}{cl} {\!\!~}^{\mathrm{G}}{\!}w & (\text{grid} (k,l): cell grid) \\ 0 & (\text{grid} (k,l): \text{medium grid}) \\ 0 & (\text{grid} (k,l): \text{wall grid}) \end{array} \right. \end{array} $$

where ^M^*w* and ^G^*w* are diffusion constants. Here, we note that the cells are in a three dimensional condition of cultured dish. The volume of medium is much larger than the total volume of those of cells attached to the bottom of the dish, so the diffusion constant of the MDP in a cell grid was set to the same value as that for a medium grid. The diffusion-direction constants show the direction of intercellular signaling (red and blue arrows in Fig. 2). When the grid (*k*,*l*) is a cell grid, ^M^*wk*,*l*′ and ^G^*wk*,*l*′ are set as diffusion coefficients leading signals in (*i*,*j*) to grid (*k*,*l*). When the grid (*k*,*l*) is a medium grid, ^G^*wk*,*l*′ is set to “0”, indicating that the signal through the GJP does not diffuse into medium grid (*k*,*l*). In contrast, the signal through the MDP diffuses into grid (*k*,*l*). There is, of course, also no diffusion into wall grids.

The quantities of the virtual signals through the MDP and the GJP are described by *M*_*i*,*j*_(*t*) and *G*_*i*,*j*_(*t*), respectively (red and blue circles in Fig. 2). Based on Eqs. (3–5), *M*_*i*,*j*_(*t*) and *G*_*i*,*j*_(*t*) in (*i*,*j*) at *t* are given by

(6) $$\begin{array}{@{}rcl@{}} M_{i,j}(t+{\Delta}t) & = & {\!\!~}^{\mathrm{M}}{\!}{\alpha}R_{i,j}(t)-{\!\!~}^{\mathrm{M}}{\!}{\beta}M_{i,j}(t)+M_{i,j}(t)  \\ &\quad&+\;\frac{{\Delta}t}{4d^{2}} \left[2\sum_{k_{1},l_{1}}{\!\!~}^{\mathrm{M}}{\!}w'_{k_{1},l_{1}}(M_{k_{1},l_{1}}(t)-M_{i,j}(t)) \right. \\[-2pt] &\quad &\left.+\sum_{k_{2},l_{2}}{\!\!~}^{\mathrm{M}}{\!}w'_{k_{2},l_{2}}(M_{k_{2},l_{2}}(t)-M_{i,j}(t))\vphantom{\frac{1}{2}}\right]\\  \end{array} $$

(7) $$\begin{array}{@{}rcl@{}} G_{i,j}(t+{\Delta}t) & = & {\!\!~}^{\mathrm{G}}{\!}{\alpha}R_{i,j}(t)-{\!\!~}^{\mathrm{G}}{\!}{\beta}G_{i,j}(t)+G_{i,j}(t)  \\[-2pt] &\quad&+\;\frac{{\Delta}t}{4d^{2}}\left[2\sum_{k_{1},l_{1}}{\!\!~}^{\mathrm{G}}{\!}w'_{k_{1},l_{1}}(G_{k_{1},l_{1}}(t)-G_{i,j}(t))  \right.\\[-2pt] &\quad&+\left. \sum_{k_{2},l_{2}}{\!\!~}^{\mathrm{G}}{\!}w'_{k_{2},l_{2}}(G_{k_{2},l_{2}}(t)-G_{i,j}(t))\right] \end{array} $$

where ^M^*α* and ^G^*α* are signal-production constants, and ^M^*αR*_*i*,*j*_(*t*) and ^G^*αR*_*i*,*j*_(*t*) are quantities of the produced virtual signals through the MDP and the GJP, respectively. *R*_*i*,*j*_(*t*) is the absorbed dose calculated based on the number of radiation tracks (Eq. (1)). The radiation track produces constant quantities of virtual signals represented by a “unit”. The parameters ^M^*β* and ^G^*β* are decay constants, and ^M^*βM*_*i*,*j*_(*t*) and ^G^*βG*_*i*,*j*_(*t*) are reductions in quantities of virtual signals, for the MDP and the GJP, respectively.

### Production of DSBs

DNA DSBs are one type of severe damage induced by irradiation and causes cell-cycle arrest or cell death. Bystander signals are also known to induce DSBs. Several studies have shown that the number of DSBs is an important factor in cell-cycle modification. For example, when the number of DSBs is smaller than a certain threshold, cells are released from G2 arrest [29]. To determine the cellular response to irradiation and also bystander signals, we estimated the number of DSBs. We made two assumptions to model DSB production: (1) the radiation and the bystander signals induce DSBs independently; (2) the number of produced DSBs is proportional to the quantities of bystander signals. It is known that the number of DSBs is proportional to the absorbed radiation dose [30]. Because the relationship between the quantity of signal and DSB production has not been clarified, we assumed a linear relationship. Based on assumption (1), the number of DSBs, *Z*_*i*,*j*_(*t*), is represented by

(8) $$\begin{array}{@{}rcl@{}} Z_{i,j}(t+{\Delta}t) & = & Z_{i,j}(t) + {\!\!~}^{\mathrm{R}} Z_{i,j}(t)+{\!\!~}^{\mathrm{M}} Z_{i,j}(t)+{\!\!~}^{\mathrm{G}}Z_{i,j}(t)  \\ &\quad+& {\!\!~}^{\mathrm{B}}Z_{i,j}(t)-{\!\!~}^{\mathrm{r}}Z_{i,j}(t) \end{array} $$

where the number of DSBs induced by radiation, virtual signals through the MDP and the GJP are ^R^*Z*_*i*,*j*_(*t*), ^M^*Z*_*i*,*j*_(*t*) and ^G^*Z*_*i*,*j*_(*t*), respectively. The number of DSBs endogenously induced by background factors such as reactive oxygen species is represented as ^B^*Z*_*i*,*j*_(*t*) [31] and the number of repaired DSBs is represented as ^r^*Z*_*i*,*j*_(*t*).

Since low-absorbed-dose irradiation induces a small number of DSBs [32], the distributions of ^R^*Z*_*i*,*j*_(*t*), ^M^*Z*_*i*,*j*_(*t*), ^G^*Z*_*i*,*j*_(*t*), and ^B^*Z*_*i*,*j*_(*t*) are based on Poisson distributions. The distribution of ^R^*Z*_*i*,*j*_(*t*) is

(9) $$\begin{array}{@{}rcl@{}} {\!\!~}^{\text{ZR}}P_{i,j} \left({\!\!~}^{\text{ZR}}n\right) &=& \frac{\left({\!\!~}^{\text{ZR}}a_{i,j}(t)\right)^{{\!\!~}^{\text{ZR}}n}e^{-{\!\!~}^{\text{ZR}}a_{i,j}(t)}}{{\!\!~}^{\text{ZR}}n!} \end{array} $$

(10) $$\begin{array}{@{}rcl@{}} {\!\!~}^{\text{ZR}}a_{i,j}(t) &=& {\!\!~}^{\text{ZR}}{\lambda}_{i,j}R_{i,j}(t) \end{array} $$

where ^ZR^*P*_*i*,*j*_(^ZR^*n*) is the probability of ^ZR^*n* DSBs induced by radiation arising in a cell over an interval *Δt*. ^ZR^*a*_*i*,*j*_(*t*) is the average of ^R^*Z*_*i*,*j*_(*t*), and is calculated as ^ZR^*λ*_*i*,*j*_*R*_*i*,*j*_(*t*) because of the proportional relationship between the number of DSBs and the absorbed radiation dose [30]. Here, ^ZR^*λ*_*i*,*j*_ is the induction coefficient for DSBs induced by irradiation.

Similarly the distributions of ^M^*Z*_*i*,*j*_(*t*) and ^G^*Z*_*i*,*j*_(*t*) are given by

(11) $$\begin{array}{@{}rcl@{}} {\!\!~}^{\text{ZM}}P_{i,j} \left({\!\!~}^{\text{ZM}}n\right) &=& \frac{\left({\!\!~}^{\text{ZM}}a_{i,j}(t)\right)^{{\!\!~}^{\text{ZM}}n}e^{-{\!\!~}^{\text{ZM}}a_{i,j}(t)}}{{\!\!~}^{\text{ZM}}n!}  \end{array} $$

(12) $$\begin{array}{@{}rcl@{}} {\!\!~}^{\text{ZM}}a_{i,j}(t) &=& {\!\!~}^{\text{ZM}}{\lambda}_{i,j}M_{i,j}(t){\Delta}t  \end{array} $$

(13) $$\begin{array}{@{}rcl@{}} {\!\!~}^{\text{ZG}}P_{i,j} \left({\!\!~}^{\text{ZG}}n\right) &=& \frac{\left({\!\!~}^{\text{ZG}}a_{i,j}(t)\right)^{{\!\!~}^{\text{ZG}}n}e^{-{\!\!~}^{\text{ZG}}a_{i,j}(t)}}{{\!\!~}^{\text{ZG}}n!}  \end{array} $$

(14) $$\begin{array}{@{}rcl@{}} {\!\!~}^{\text{ZG}}a_{i,j}(t) &=& {\!\!~}^{\text{ZG}}{\lambda}_{i,j}G_{i,j}(t){\Delta}t \end{array} $$

where ^ZM^*P*_*i*,*j*_(^ZM^*n*) is the probability of ^ZM^*n* DSBs induced by the MDP arising in a cell over interval *Δt*. ^ZG^*P*_*i*,*j*_(^ZG^*n*) is the probability of ^ZG^*n* DSBs induced by the GJP arising in a cell over *Δt*. ^ZM^*a*_*i*,*j*_(*t*) and ^ZG^*a*_*i*,*j*_(*t*) are the averages of ^M^*Z*_*i*,*j*_(*t*) and ^G^*Z*_*i*,*j*_(*t*), and are calculated as ^ZM^*λ*_*i*,*j*_*M*_*i*,*j*_(*t*)*Δt* and ^ZG^*λ*_*i*,*j*_*G*_*i*,*j*_(*t*)*Δt*, respectively, based on assumption (2). Here, ^ZM^*λ*_*i*,*j*_ and ^ZG^*λ*_*i*,*j*_ are induction coefficients for DSBs induced by virtual signals through the MDP and the GJP, respectively.

The distribution of ^B^*Z*_*i*,*j*_(*t*) is given by

(15) $$\begin{array}{@{}rcl@{}} {\!\!~}^{\text{ZB}}P_{i,j}\left({\!\!~}^{\text{ZB}}n\right) & = & \frac{\left({\!\!~}^{\text{ZB}}a_{i,j}\right)^{{\!\!~}^{\text{ZB}}n}e^{-{\!\!~}^{\text{ZB}}a_{i,j}}}{{\!\!~}^{\text{ZB}}n!}.  \end{array} $$

(16) $$\begin{array}{@{}rcl@{}} {\!\!~}^{\text{ZB}}a_{i,j} &=& {\!\!~}^{\text{ZB}}{\lambda}_{i,j} \end{array} $$

where ^ZB^*P*_*i*,*j*_(^ZB^*n*) is the probability of ^ZB^*n* DSBs induced by background factors arising in a cell over *Δt*. ^ZB^*a*_*i*,*j*_ is the average of ^B^*Z*_*i*,*j*_(*t*) and ^ZB^*λ*_*i*,*j*_ is the corresponding induction coefficient.

The number of repaired DSBs, ^r^*Z*_*i*,*j*_(*t*), is calculated using a probability of DSB repair. Most of the produced DSBs are known to be repaired in living cells through several independent pathways. Radiation-induced DSBs are also thought to have various complexities [33] and to be repaired with different kinetics in each pathway. Although some repair kinetics models have been reported in previous studies [34, 35], there is little experimental evidence that reaction rate constants are completely discriminated for each pathway. The relationship between DSB complexity and repair pathway has also not been elucidated. Hence, in the current stage of our model, we assumed for simplicity that all DSBs are repaired with the same probability, ^Zr^*λ*_*i*,*j*_.

**Fig. 3:**
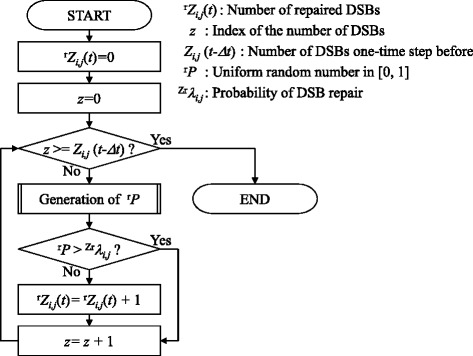
Flowchart of algorithm to determine the number of repaired double-strand breaks (DSBs). All DSBs are repaired with the same probability, ^Zr^ *λ*_*i*,*j*_

The calculation algorithm for ^r^*Z*_*i*,*j*_(*t*) is shown in Fig. 3. First, ^r^*Z*_*i*,*j*_(*t*) is set to 0. The index *z* in the algorithm (Fig. 3) counts the number of DSBs, and is initially set to 0. When *Z*_*i*,*j*_(*t*−*Δt*) is larger than *z*, a uniform random number, ^r^*P*, in [0,1], is generated. When ^r^*P*> ^Zr^*λ*_*i*,*j*_, the DSB is not repaired. When ^r^*P* is smaller than ^Zr^*λ*_*i*,*j*_, the DSB is repaired and ^r^*Z*_*i*,*j*_(*t*) is increased by one. After the comparison between ^r^*P* and ^Zr^*λ*_*i*,*j*_, *z* is increased by one. The generation of ^r^*P* and the comparison are repeated until *z* reaches *Z*_*i*,*j*_(*t*−*Δt*).

The parameters ^ZR^*λ*_*i*,*j*_, ^ZM^*λ*_*i*,*j*_, ^ZG^*λ*_*i*,*j*_, ^ZB^*λ*_*i*,*j*_, and ^Zr^*λ*_*i*,*j*_ are initially set to different values for individual grids. To reflect the characteristics of individual cells, we assume that the parameters are taken from the positive part of a normal distribution.

### Cellular response

Cell-cycle arrest is known to occur at certain checkpoints when DNA is damaged, and modification of the cell cycle is an important index to measure when monitoring radiation-induced responses. However, radiation-induced cellular responses have been estimated mainly based on cell death so far. In our model, we consider both cell cycle progression and cell death after irradiation.

**Fig. 4:**
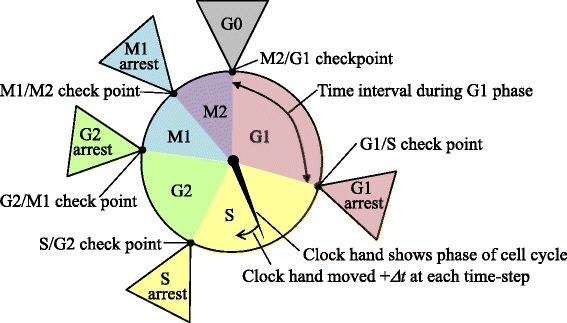
Virtual clock to describe cell-cycle progression and cell-cycle arrest. The virtual clock consists of five phases: G1, S, G2, M1, and M2, and five checkpoints. Each checkpoint has a cell-cycle arrest phase. The clock hand shows the phase of the cell cycle and moves *Δ* *t* at each time step

The phase of the cell cycle or cell death for the cell grid (*i*,*j*) at time *t* is represented by *S*_*i*,*j*_(*t*). The progress or arrest of the cell cycle is determined by a virtual clock, as shown in Fig. 4. The virtual clock is divided into five phases, G1, S, G2, M1, and M2. M1, and M2 are a substructure of the M phase, which is divided at the M1/M2 checkpoint. The checkpoints (G1/S, S/G2, G2/M1, and M1/M2) are the time points defined by the clock. The time of cell division is represented by the M2/G1 time point. *S*_*i*,*j*_(*t*) is determined by the position of the virtual clock hand. When the clock hand stops at each checkpoint, *S*_*i*,*j*_(*t*) indicates cell-cycle arrest.

Cell death is generally divided into reproductive death [36] and interphase death [37]. Reproductive death is the loss of the proliferative ability of the cell, and cells keep their cellular activity even after stopping cell division. Interphase death shows no proliferation, and the cells are disrupted. We modeled both types of cell death, taking into account that the reproductively dead cells still transfer signals through the GJP.

**Fig. 5:**
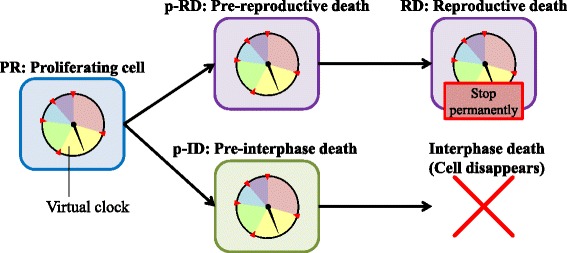
State transitions from proliferation to cell death. Four cellular states were considered: the proliferating (PR) state; the pre-reproductive death (p-RD) state; the reproductive death (RD) state; and the pre-interphase death (p-ID) state. The black arrows represent the direction of state transition. Each state has a virtual clock

Cellular states are represented by four states, the proliferating (PR), pre-reproductive death (p-RD), reproductive death (RD), and pre-interphase death (p-ID) states, as shown in Fig. 5. Each state has a virtual clock. We used *S*_*i*,*j*_(*t*) to represent not only the phase of the cell cycle but also these four states. In the PR state, the cells have infinite proliferative capacity. In the p-RD state, the number of cell divisions is randomly assigned from one to three. When the cell division stops in the p-RD state, the cell transits to the RD state, in which the cell remains but the virtual clock stops permanently. In the p-ID state, the number of cell divisions is less than one and so the cell disappears. The transition pathways from the PR state to the p-RD or p-ID states are assumed to be irreversible.

**Fig. 6:**
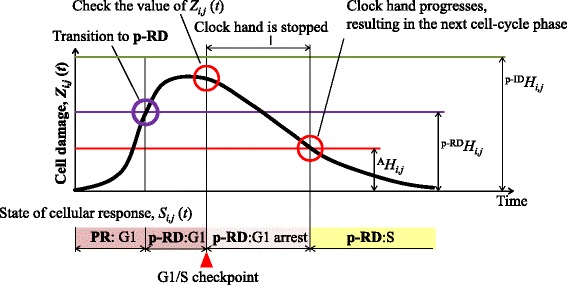
State transition based on the number of DSBs. An example of the time course of the number of DSBs, *Z*_*i*,*j*_(*t*), and the state transition. *Z*_*i*,*j*_(*t*) increases with time, and then decreases. According to *Z*_*i*,*j*_(*t*), the cellular state *S*_*i*,*j*_(*t*) is a cell-cycle phase or cell death

It has been reported that the G2/M checkpoint has a threshold in the number of DSBs to determine the cell-cycle progression or arrest [29]. In our model, we set threshold values using the number of DSBs, *Z*_*i*,*j*_(*t*). The threshold for transition between the arrest and the progression of a cell cycle was represented by ^A^*H*_*i*,*j*_. The thresholds for transition from the PR to the p-RD state was represented by ^p−RD^*H*_*i*,*j*_, and that for transition from the PR to the p-ID state by ^p−ID^*H*_*i*,*j*_. The state of the cell, *S*_*i*,*j*_(*t*), is in progression or is arrested when *Z*_*i*,*j*_(*t*) lies below or exceeds the threshold values for each phase of the cell cycle. Figure 6 schematically shows the threshold values determining the state transitions. When *Z*_*i*,*j*_(*t*) is larger than the threshold, ^p−RD^*H*_*i*,*j*_, for transition from the PR to the p-RD states, *S*_*i*,*j*_(*t*) takes the “p-RD:G1”. Similarly when *Z*_*i*,*j*_(*t*) is larger than the threshold, ^A^*H*_*i*,*j*_, for transition to G1 arrest, *S*_*i*,*j*_(*t*) takes the “p-RD:G1 arrest” and the clock hand stops. When *Z*_*i*,*j*_(*t*) is smaller than ^A^*H*_*i*,*j*_, *Z*_*i*,*j*_(*t*) is in the “p-RD:S” state and the clock hand is progressing.

When *S*_*i*,*j*_(*t*) is the PR or the p-RD state and reaches the M2/G1 checkpoint, the cell is divided into two daughter cells. One of the adjacent medium grids is replaced by one of the daughter cells. If there is no medium grid in the adjacent area, *S*_*i*,*j*_(*t*) takes the G0 phase.

The parameter values for state transitions ^A^*H*_*i*,*j*_, ^p−RD^*H*_*i*,*j*_, and ^p−ID^*H*_*i*,*j*_ are set differently for each individual grid. To reflect the characteristics of individual cells, we assumed that the parameters are taken from the positive part of a normal distribution. All the variable numbers and parameters used in our model are shown in Tables 1 and 2.

**Table 1:** Variable numbers

Variable number	Name
*i*	Horizontal index of grid
*j*	Vertical index of grid
*t*	Time
*K*_*i*,*j*_(*t*)	Number of radiation tracks
*R*_*i*,*j*_(*t*)	Absorbed dose
^K^ *P*_*i*,*j*_(^K^ *n*)	Probability according to a Poisson distribution when *K*_*i*,*j*_(*t*) is ^K^ *n*
(*k*_1_,*l*_1_)	Positions of four nearest-neighbor grids surrounding grids (*i*,*j*): i.e., one of (*i*+1,*j*), (*i*−1,*j*), (*i*,*j*+1), and (*i*,*j*−1)
(*k*_2_,*l*_2_)	Positions of four skew nearest-neighbor grids surrounding grid (*i*,*j*): that is, one of (*i*+1,*j*+1), (*i*−1,*j*+1), (*i*+1,*j*+1), and (*i*−1,*j*−1)
^M^ *w* *k*,*l*′	Diffusion-direction constant of virtual signal through the MDP
^G^ *w* *k*,*l*′	Diffusion-direction constant of virtual signal through the GJP
*M*_*i*,*j*_(*t*)	Quantity of the virtual signal through the MDP
*G*_*i*,*j*_(*t*)	Quantity of the virtual signal through the GJP
*Z*_*i*,*j*_(*t*)	The number of DSBs
^R^ *Z*_*i*,*j*_(*t*)	Number of DSBs induced by radiation
^M^ *Z*_*i*,*j*_(*t*)	Number of DSBs induced by the virtual signal through the MDP
^G^ *Z*_*i*,*j*_(*t*)	Number of DSBs induced by the virtual signal through the GJP
^B^ *Z*_*i*,*j*_(*t*)	Number of DSBs endogenously induced by background factors
^r^ *Z*_*i*,*j*_(*t*)	Number of repaired DSBs
^ZR^ *P*_*i*,*j*_(^ZR^ *n*)	Probability according to a Poisson distribution when ^M^ *Z*_*i*,*j*_(*t*) is ^ZR^ *n*
^ZM^ *P*_*i*,*j*_(^ZM^ *n*)	Probability according to a Poisson distribution when ^M^ *Z*_*i*,*j*_(*t*) is ^ZM^ *n*
^ZG^ *P*_*i*,*j*_(^ZG^ *n*)	Probability according to a Poisson distribution when ^G^ *Z*_*i*,*j*_(*t*) is ^ZG^ *n*
^ZB^ *P*_*i*,*j*_(^ZB^ *n*)	Probability according to a Poisson distribution when ^B^ *Z*_*i*,*j*_(*t*) is ^ZB^ *n*
*S*_*i*,*j*_(*t*)	State of cell grid, such as phase of the cell cycle or cell death

DSB: double-strand break; GJP: gap junctional pathway; MDP: medium-mediated pathway

**Table 2:** Parameters

Parameter	Name	Value used in simulations
*Δ* *t*	Time interval	0.1 s
*d*	Width of grid (grid spacing)	10 *μ*m
*D*_1track_	Expected value of the absorbed dose given by one radiation track	0.001 Gy
^K^ *a*	Average number of radiation tracks passing through a grid over *Δ* *t*	1,000 track/min
^M^ *w*	Diffusion constants of virtual signals through the MDP	1.0×10^−10^ m ^2^/s
^M^ *α*	Signal-production constant of virtual signals through the MDP	1 unit/Gy
^M^ *β*	Decay constant of virtual signals through the MDP	4.6×10^−6^ s ^−1^
^G^ *w*	Diffusion constants of virtual signals through the GJP	5.0×10^−11^ m ^2^/s
^G^ *α*	Signal-production constant of virtual signals through the GJP	1 unit/Gy
^G^ *β*	Decay constant of virtual signals through the GJP	1.18×10^−3^ s ^−1^
^ZR^ *λ*_*i*,*j*_	DSB induction coefficients for radiation	G0 or G1 phase
		AV: 40; SD: 22 DSBs/Gy
		S phase
		AV: 80; SD: 24 DSBs/Gy
		G2 phase
		AV: 80; SD: 26 DSBs/Gy
		M1 or M2 phase
		AV: 80; SD: 16 DSBs/Gy
^ZM^ *λ*_*i*,*j*_	DSB induction coefficients for virtual signals through the MDP	G0 or G1 phase
		AV: 6.0×10^−3^; SD: 1.0×10^−2^ DSBs/unit/s
		S, G2, M1, or M2 phase
		AV: 1.2×10^−2^; SD: 1.0×10^−2^ DSBs/unit/s
^ZG^ *λ*_*i*,*j*_	DSB induction coefficients for virtual signals through the GJP	G0 or G1 phase
		AV: 6.0×10^−2^; SD: 1.0×10^−2^ DSBs/unit/s
		S, G2, M1, or M2 phase
		AV: 1.2×10^−1^; SD: 1.0×10^−2^ DSBs/unit/s
^ZB^ *λ*_*i*,*j*_	DSB induction coefficients for background factors	G0 or G1 phase
		AV: 1.4×10^−5^; SD: 0 DSBs/s
		S, G2, M1, or M2 phase
		AV: 2.8×10^−5^; SD: 0 DSBs/s
^Zr^ *λ*_*i*,*j*_	Probability of DSB repair	G0 or G1 phase
		AV: 9.33×10^−7^; SD: 2.0×10^−5^ s ^−1^
		S, G2, M1, or M2 phase
		AV: 7.45×10^−7^; SD: 2.0×10^−5^ s ^−1^
^A^ *H*_*i*,*j*_	Thresholds for transition between cell-cycle progression and cell-cycle arrest	G1/S checkpoint
		AV: 5; SD: 1 DSBs
		S/G2 checkpoint
		AV: 40; SD: 8 DSBs
		G2/M1 checkpoint
		AV: 20; SD: 4 DSBs
		M1/M2 checkpoint
		AV: 40; SD: 8 DSBs
^p−RD^ *H*_*i*,*j*_	Thresholds for transition from the PR to the p-RD state	G0 or G1 phase
		AV: 93; SD: 38 DSBs
		S phase
		AV: 42; SD: 144 DSBs
		G2 phase
		AV: 116; SD: 124 DSBs
		M1 or M2 phase
		AV: 22; SD: 91 DSBs
^p−ID^ *H*_*i*,*j*_	Thresholds for transition from the PR to the p-ID state	G0 or G1 phase
		AV: 186; SD: 38 DSBs
		S phase
		AV: 84; SD: 144 DSBs
		G2 phase
		AV: 232; SD: 124 DSBs
		M1 or M2 phase
		AV: 44; SD: 91 DSBs
*T* ^c^	Period of each phase of the cell cycle	G1 phase
		AV: 11; SD: 2.2 h
		S phase
		AV: 8; SD: 1.6 h
		G2 phase
		AV: 4; SD: 0.8 h
		M1 or M2 phase
		AV: 0.5; SD: 0.1 h

AV: average; SD: standard deviation

## Parameter estimation

To simulate individual cellular responses by bystander signals, our model needs to set the values of the 19 parameters shown in Table 2. Some of these values can be found from previous studies. We estimated the others using experimental data as follows.

**Fig. 7:**
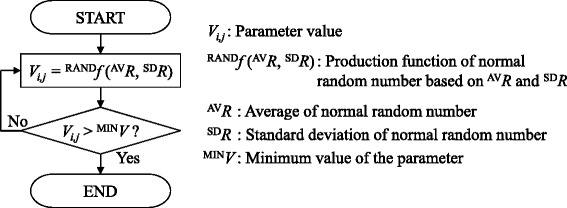
Flowchart of the individual parameter setting method. The value of a parameter is set based on a truncated normal distribution that has a minimum value

As mentioned above, some of parameters have a distribution which was generated by the algorithm in Fig. 7. First, a parameter value, *V*_*i*,*j*_, is generated using a function for producing a normal random number, ^RAND^*f*(^AV^*R*, ^SD^*R*), where ^AV^*R* and ^SD^*R* are the average and standard deviation of the normal random number. Next, *V*_*i*,*j*_ is compared with the minimum value of the distribution, ^MIN^*V*. Until the condition *V*_*i*,*j*_> ^MIN^*V* is satisfied, *V*_*i*,*j*_ is regenerated by ^RAND^*f*(^AV^*R*, ^SD^*R*). The parameter values of all grids were determined using this method. To estimate ^AV^*R* and ^SD^*R* for each parameter, we fitted the calculated data to selected experimental data. For the calculation of cell-cycle modification by bystander effects, there is no parameter set experimentally obtained from one cell line. Thus, we assembled parameter values from various cell lines used in previous studies.

### Change in the number of DSBs with time

To estimate ^AV^*R* and ^SD^*R* for the probability of DNA repair, ^Zr^*λ*_*i*,*j*_, data calculated by our model were fitted to a time course of the number of DSBs determined by the number of *γ*-H2AX foci per cell after X-ray irradiation [29]. The experimental data showed that the number of DSBs after irradiation (1.5 Gy) decreased with time.

**Fig. 8:**
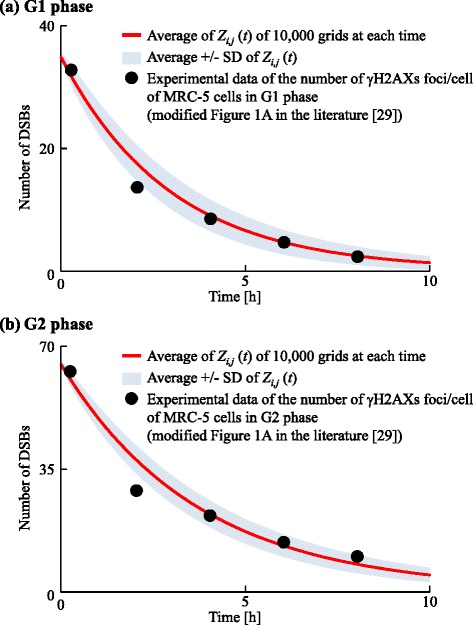
Data fitting for the change of the number of DSBs with time. The red line represents the average number of DSBs, *Z*_*i*,*j*_(*t*). Black points represent the experimental data of the number of gamma-H2AXs foci/cells or MRC-5 cells in the G1 or G2 phases (modified from Fig. 1a of [29]). We calculated *Z*_*i*,*j*_(*t*) for (**a**) the G1 phase and (**b**) the G2 phase

We calculated the number of DSBs using Eq. (8). So many DSBs were produced by irradiation in the experiment that the number of DSBs caused by background factors or bystander signals could be neglected. Thus, the number of DSBs induced by virtual signals through the MDP, ^M^*Z*_*i*,*j*_(*t*), and through the GJP, ^G^*Z*_*i*,*j*_(*t*), were both set to 0. The experimental data used for data fitting (shown in Fig. 8a) indicated that the number of DSBs just after (5 min) irradiation (1.5 Gy) was 30–35 [29]. We therefore set ^R^*Z*_*i*,*j*_(*t*=0)=35. Because the number of DSBs endogenously induced by background factors, ^B^*Z*_*i*,*j*_(*t*), was much smaller than ^R^*Z*_*i*,*j*_(0), ^B^*Z*_*i*,*j*_(*t*) was set to 0. To calculate the average number of DSBs, we used 10,000 grids. We performed a preliminary search and found the ranges of parameter values that could reproduce the experimental data. The ranges for ^AV^*R* and ^SD^*R* were set to be from 1.0×10^−8^ to 1.0×10^−6^ and from 0 to 1.0×10^−4^, respectively. We determined the values of ^AV^*R* and ^SD^*R* of ^Zr^*λ*_*i*,*j*_ in the G1 phase by data fitting to be 9.33×10^−7^ s ^−1^ and 2.0×10^−5^ s ^−1^, respectively, and the results are shown in Fig. 8a.

In a similar way, ^AV^*R* and ^SD^*R* for ^Zr^*λ*_*i*,*j*_ in the G2 phase were estimated to be 7.45×10^−7^ s ^−1^ and 2.0×10^−5^ s ^−1^, respectively. Calculation data fitted to experimental data are shown in Fig. 8b.

### Number of cells that have DSBs following intercellular signaling

To estimate ^AV^*R* and ^SD^*R* for the induction coefficients for DSBs induced by virtual signals and background factors, ^ZM^*λ*_*i*,*j*_, ^ZG^*λ*_*i*,*j*_, and ^ZB^*λ*_*i*,*j*_, results calculated using our model were fitted to the number of bystander cells with DSBs after *α*-irradiation [27]. Since the irradiated dishes in the experiment were filled with cells in the G0 phase, we used a space composed of 100 ×100 grids in the G0 phase. The grid width, *d*, was set to 10 *μ*m.

**Fig. 9:**
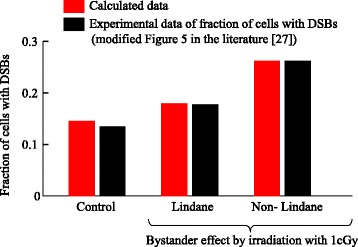
Data fitting for the number of cells with DSBs. The red and black bars represent calculated data and experimental data (modified from Fig. 5 of [27]), respectively. Lindane has been used as an inhibitor of intercellular signaling through the GJP. The calculation for lindane treatment shows the bystander effect from the MDP. The calculation for non-lindane treatment shows the bystander effect from both the MDP and the GJP

First, we estimated ^AV^*R* for ^ZB^*λ*_*i*,*j*_ using control data in the experiment [27]. The ^SD^*R* of ^ZB^*λ*_*i*,*j*_ was set to 0 since the standard deviation is much smaller than the average. Because the control data have been measured under sham-irradiation conditions, the absorbed radiation dose and the quantities of virtual signals through the MDP and the GJP, *R*_*i*,*j*_(*t*), *M*_*i*,*j*_(*t*), and *G*_*i*,*j*_(*t*), were set to 0. We then used Eqs. (8) and (15) to calculate the number of DSBs, *Z*_*i*,*j*_(*t*). The probability of DNA repair, ^Zr^*λ*_*i*,*j*_, was set to the value for the G0 phase estimated by the method described in the previous section. In the previous report, cells with one or more *γ*-H2AX foci were scored as cells with DSBs [27]. Based on this experimental method, we calculated *Z*_*i*,*j*_(*t*) in 10,000 grids over 30 min, then the number of cells that had *Z*_*i*,*j*_(*t*)>0 were counted as cells with DSBs. The range of searching for the value of ^AV^*R* for ^ZB^*λ*_*i*,*j*_ was set to be from 5.0×10^−6^ s ^−1^ to 1.5×10^−5^ s ^−1^. We determined values of ^AV^*R* for ^ZB^*λ*_*i*,*j*_ in the G0 phase by data fitting, and the results are shown in Fig. 9. The value of ^AV^*R* was estimated to be 1.4×10^−5^ s ^−1^. The value of ^AV^*R* for ^ZB^*λ*_*i*,*j*_ in the G1 phase was set to be the same as in the G0 phase. The values of ^AV^*R* for ^ZB^*λ*_*i*,*j*_ in the S, G2, M1, and M2 phases were set to 2.8×10^−5^ s ^−1^ because the amount of DNA in these phases is double that in the G1 phase.

For ^ZM^*λ*_*i*,*j*_, we used the data [27] of cells treated with lindane which was used as an inhibitor of gap-junctional intercellular signaling, and the virtual signal through the GJP, *G*_*i*,*j*_(*t*), as well as the absorbed dose, *R*_*i*,*j*_(*t*), were set to 0. Then we used Eqs. (6), (8), (11), (12) and (15) to calculate the number of DSBs, *Z*_*i*,*j*_(*t*). We assumed that the virtual signal through the MDP had the same characteristics as those for cytokines, such as interleukin 8. The signal-production constant of the virtual signal through the MDP, ^M^*α*, was set to 1 unit/Gy where “unit” denotes an arbitrary quantity of the virtual signal. Because the bystander signals through the MDP were still active at 60 hours after irradiation [3], the decay constant of the virtual signal through MDP, ^M^*β*, was set to 4.6×10^−6^ s ^−1^. The diffusion constant of the virtual signal through the MDP was set to 1.0×10^−10^ m ^2^/s [38]. The initial value of virtual signals through the MDP, *M*_*i*,*j*_(*t*), in all cell grids was set to 2.5×10^−3^ units based on the radiation dose and the number of irradiated cells in the experiment. The values of ^ZB^*λ*_*i*,*j*_ and ^Zr^*λ*_*i*,*j*_ were set to the estimates shown in Table 2. The ranges of searching for the values of ^AV^*R* and ^SD^*R* were set to be from 2.0×10^−3^ to 4.0×10^−3^ DSBs/unit/s and from 0 to 1.0×10^−4^ DSBs/unit/s, respectively. We determined the values of ^AV^*R* and ^SD^*R* by data fitting, and the results are shown in Fig. 9. The values of ^AV^*R* and ^SD^*R* were estimated to be 6.0×10^−3^ DSBs/unit/s and 1.0×10^−2^ DSBs/unit/s, respectively.

For ^ZG^*λ*_*i*,*j*_, we used data obtained at 30 min after 1 cGy-irradiation [27] and used Eqs. (6–8) and (11–15) to calculate the number of DSBs, *Z*_*i*,*j*_(*t*). As mentioned above, the virtual signal through the GJP was assumed to have the characteristics of calcium ions. The signal-production constant of the virtual signal through the GJP, ^G^*α*, was set to 1 unit/Gy. The decay constant, ^G^*β*, was set based on the efflux of calcium ions from the cell. The velocity of the efflux of calcium ions could be divided into a fast phase and a slow phase [39]. However, the life time of the fast phase signal was too short to affect cellular responses ranging from minutes to hours, so we ruled out this phase. The value of ^G^*β* was set to 1.18×10^−3^ s^−1^ based on the rate constant of the slow phase. The diffusion constant of the virtual signal through the GJP was set to 5.0×10^−11^ m ^2^/s, consistent with reported values [40]. The values of ^ZM^*λ*_*i*,*j*_, ^ZB^*λ*_*i*,*j*_, and ^Zr^*λ*_*i*,*j*_ were set to the estimates shown in Table 2. The initial values of *M*_*i*,*j*_(*t*) and *G*_*i*,*j*_(*t*) were set to 2.5×10^−3^ units. The ranges for searching for the values of ^AV^*R* and ^SD^*R* were from 2.0×10^−3^ to 8.0×10^−2^ DSBs/unit/s and from 0 to 1.0×10^−4^ DSBs/unit/s, respectively. We determined the values of ^AV^*R* and ^SD^*R* by data fitting, and the results are shown in Fig. 9. The values ^AV^*R* and the ^SD^*R* were finally estimated to be 6.0×10^−2^ DSBs/unit/s and 1.0×10^−2^ DSBs/unit/s, respectively.

### Cell survival fractions

The values of ^AV^*R* and ^SD^*R* for the induction coefficient for DSBs induced by radiation, ^ZR^*λ*_*i*,*j*_, and the threshold of the DSB number for transition from the PR to the p-RD state, ^p−RD^*H*_*i*,*j*_, were estimated based on cell survival fraction data. A cell grid in the PR state was defined to be equivalent to a surviving cell observed in experiments.

**Fig. 10:**
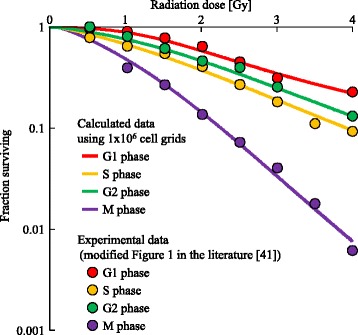
Data fitting for the cell survival fractions. Lines represent calculated data. Points represent experimental data (modified from Fig. 1 of [41]). Different colors indicate each cell-cycle phase when cells were irradiated with doses of 0.5–4 Gy

The survival of human cancer cells (HeLa cells) is the only data to include survival fractions for each cell-cycle phase [41]. The parameter setting and the number of grids were determined in the same way as in the previous subsection. We used Eqs. (1–2) and (8–10) to calculate the number of DSBs, *Z*_*i*,*j*_(*t*). The expected value of the absorbed dose given by one radiation track, *D*_1track_, was set to 1.0×10^−3^ Gy. The dose rate was set to 0.5 Gy/min. The irradiation time was set to 1, 2, 3, 4, 5, 6, 7, or 8 min according to the radiation doses in the experiment. The values of ^AV^*R* for ^ZR^*λ*_*i*,*j*_ in the G0 and G1 phases were set to 40 DSBs/Gy because a previous study estimated that 1 Gy irradiation induced 40 DSBs [42]. Since the amount of DNA in the S, G2, and M phases is double that in the G1 phase, the values of ^AV^*R* in the S, G2, M1, and M2 phases were set to 80 DSBs/Gy. During the irradiation period, the number of induced DSBs was much larger than that of repaired DSBs. Thus, ^r^*Z*_*i*,*j*_(*t*) was set to 0. We calculated the number of DSBs, *Z*_*i*,*j*_(*t*), in 10,000 grids. The number of surviving cells was defined as the number of grids that had *Z*_*i*,*j*_(*t*)< ^p−RD^*H*_*i*,*j*_. In a similar way to the parameter estimation in previous sections, we determined the value of ^SD^*R* for ^ZR^*λ*_*i*,*j*_, and the values of ^AV^*R* and ^SD^*R* for ^p−RD^*H*_*i*,*j*_ by data fitting. The results are shown in Fig. 10. The determined values of ^AV^*R* and ^SD^*R* are shown in Table 2.

## Results

### Simulation setup

To analyze the dynamics and general properties of our model, we examined cell-cycle modification caused by intercellular signaling after exposure to various doses of radiation.

**Fig. 11:**
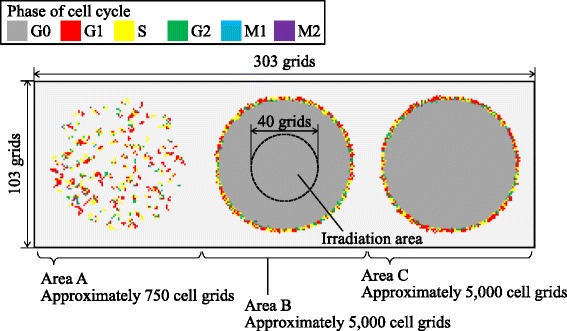
Schematic of simulation conditions. The simulation space is composed of 103 ×303 grids. Different colors represent the cell-cycle phase. The space is divided into three areas, A, B, and C, and cellular populations are introduced in each area. Only the central area of the population in area B was irradiated

We used a simulation space composed of 103 ×303 grids, as shown in Fig. 11. The space was divided into three areas, A, B, and C, and cellular populations were introduced in each area. The cells in area A were cultured sparsely: cells occupied 15 % of a circle with a radius of 40 grids. In areas B and C, the cell populations were almost at 100 % confluence. Since the cells in the area A had enough space to progress their cell cycle for growth, the effect on cell-cycle modification is expected to be more easily observed in area A than in the other areas. In area B, only the central area of the population was irradiated. The non-irradiated cells received intercellular signals through both the MDP and the GJP, on the other hand the cells in areas A and C received signals through the MDP only. The contribution of the GJP to cell-cycle modification could be examined by comparing the populations in areas B and C.

Within the irradiation area, cells were irradiated uniformly (*D*_1track_=1.0×10^−3^ Gy). The dose rate was set at 1 Gy/min and the tested doses were 0, 0.1, 0.2, 0.5, 1, 2, or 5 Gy. The case without exposure (0 Gy) was the control.

For the virtual signals through the MDP and the GJP, the parameters shown in Table 2 were used.

The number of DSBs that induced G2/M checkpoint release has been suggested to be approximately 20 [29]. Thus, the average threshold, ^A^*H*_*i*,*j*_, at G2/M1 checkpoints was set to 20 DSBs. The average thresholds were set to 5 and 40 DSBs for G1/S and S/G2, respectively (Shibata A., private communication, 2015). The M1/M2 checkpoint is the spindle checkpoint and does not directly relate to DSBs. Thus, the threshold value was set to 40, which was the largest value used in the present study. Standard deviations of the thresholds were set to 20 % of the averages.

For the averages and standard deviations of thresholds for transition from the PR to the p-RD state, ^p−RD^*H*_*i*,*j*_, we used the values estimated by data fitting shown in Table 2.

The threshold of interphase death was determined to realize a high frequency of death in a higher dose region and low frequency in a lower dose region, as reported by Puck and Marcus [43]. We assumed that the threshold, ^p−ID^*H*_*i*,*j*_, was two times larger than the threshold for reproductive death based on the experimental frequencies of the two types of cell death.

An average cell cycle period was set to 24 h based on HeLa cell data [44]. The periods of the G1, S, G2, M1, and M2 phases were set to 11, 8, 4, 0.5, and 0.5 h, respectively. The calculated cell culture time was set to 96 h to simulate cell fates even when the cycles were greatly prolonged.

We performed 10 trials using various initial conditions for the positions of cell grids and random numbers for the calculation of Poisson distributions.

### Intercellular signaling

**Fig. 12:**
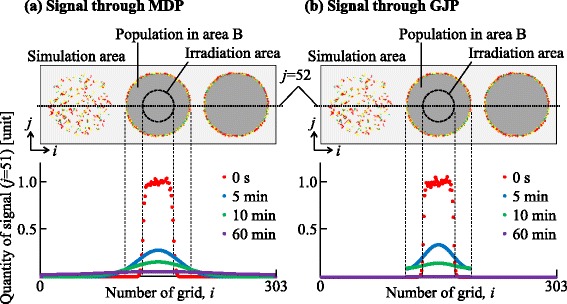
Quantities of virtual signals. **a** Quantity of signal through the MDP, *M*_*i*,*j*_(*t*), in grids (*i*=1,2,…,303,*j*=52). **b** Quantity of signal through the GJP, *G*_*i*,*j*_(*t*) in grids (*i*=1,2,…,303,*j*=52). The horizontal axis indicates the value of *i*. The top panel shows a schematic layout of the simulation space. Each color represents a different time after irradiation

The quantities of virtual signals in grids (*i*=1, 2,…,303,*j*=52) for 0.1 Gy irradiation are shown in Fig. 12, indicating the quantity of signal through the MDP, *M*_*i*,*j*_(*t*), (Fig. 12a), and through the GJP, *G*_*i*,*j*_(*t*) (Fig. 12b). At *t*=0, quantities of virtual signals at the irradiation area were around 1 unit. After 5 min, *M*_*i*,52_(*t*) for the population in area B was smaller than *G*_*i*,52_(*t*), reflecting the size of the diffusion area. After 10–60 min, the virtual signals through the MDP were transmitted to distant grids and finally diffused into the whole area. On the other hand those through the GJP were transmitted within area B, and finally almost disappeared. These results indicate that non-irradiated cells could be exposed to an MDP signal over a longer period than a GJP signal.

### Dependence of cell-cycle modification on radiation dose

**Fig. 13:**
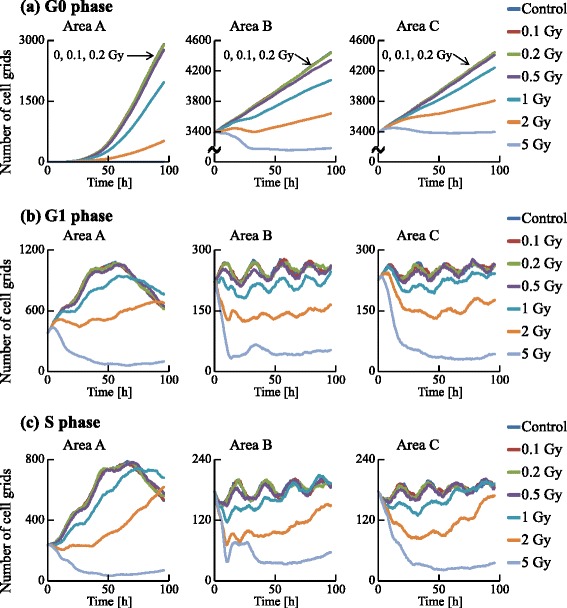
Time courses of the numbers of cells in the G0, G1, and S phases. The numbers of cell grids in the G0, G1, and S phases were counted at each time. The horizontal axis indicates the time. Each color represents a radiation dose. The result of 0 Gy irradiation was determined as a control

**Fig. 14:**
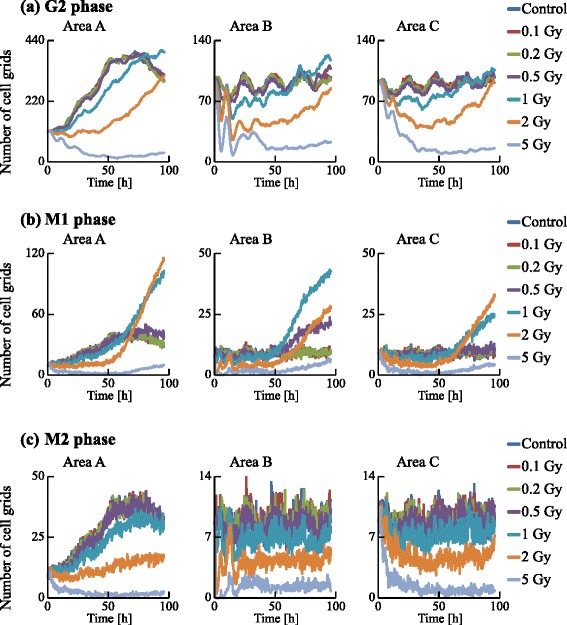
Time courses of the numbers of cells in the G2, M1, and M2 phases. The numbers of cell grids in the G2, M1, and M2 phase were counted at each time. The horizontal axis indicates the time. Each color represents a radiation dose

**Fig. 15:**
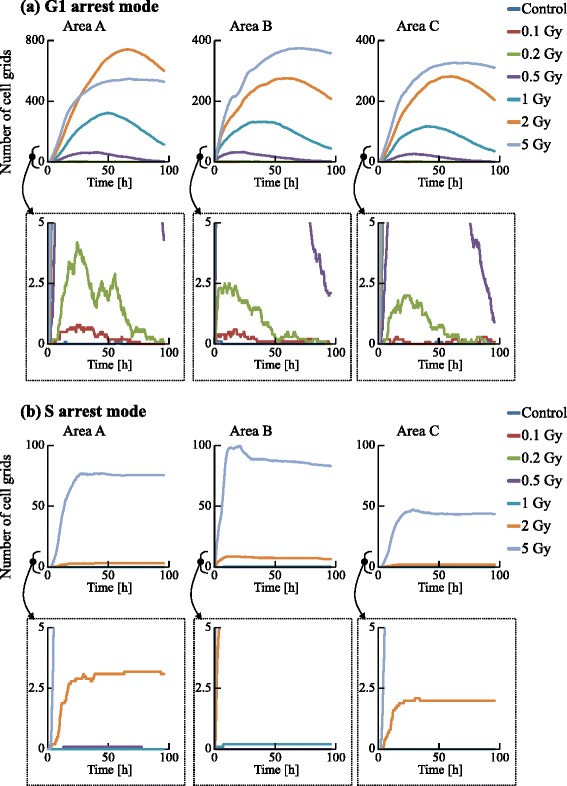
Time courses of the number of cells in the G1 and S arrest modes. The numbers of cell grids in the G1 and S arrest modes were counted at each time. The horizontal axis indicates the time. Each color represents a radiation dose

**Fig. 16:**
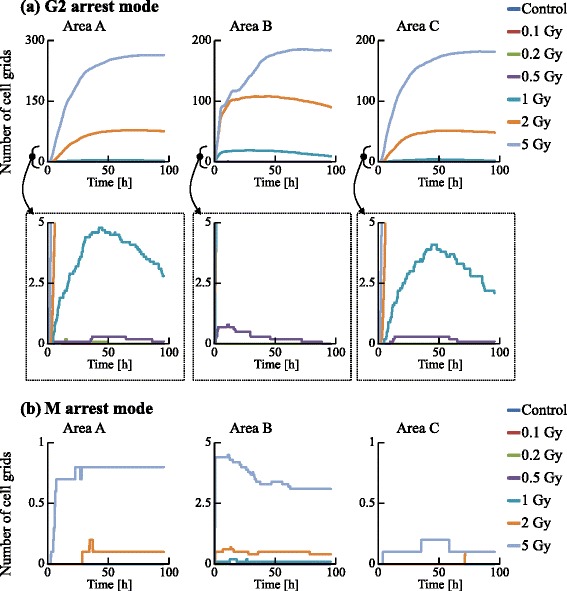
Time courses of the number of cells in the G2 and M arrest modes. The numbers of cell grids in the G2 and M arrest modes were counted at each time. The horizontal axis indicates the time. Each color represents a radiation dose

We examined the dynamics of the population in all the areas, as shown in Figs. 13 and 14. The irradiated cells were excluded from the cell counting in area B, and the cells were subtracted from the number of cells at G0 in area C to compare these two areas. Time courses for 0.1, 0.2, and 0.5 Gy irradiation were similar to that of the control. However, those for higher doses (1, 2, and 5 Gy) significantly decreased with increasing dose. The numbers of cells in each cell-cycle arrest mode after irradiation is shown in Figs. 15 and 16. In the low-dose region (0.1–0.2 Gy) there were no significant effects for all cases. However, above 0.5 Gy, the number of arrested cells increased with dose in all cases except for G1 arrest. Area A showed significant cell-cycle modification by intercellular signaling for all cases except for G0.

**Fig. 17:**
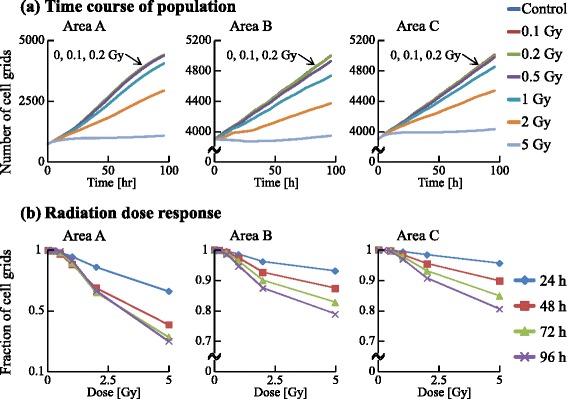
Time course and radiation dose response of cellular populations. The numbers of all cell grids were counted at each time. **a** The time course of the population in each area. The horizontal axis indicates the time. Each color represents a radiation dose. **b** The radiation dose response of the population in each area. The horizontal axis indicates the radiation dose. Each color represents time

Area A was analyzed further to investigate dose dependence. The number cells decreased with time for the G1, S, and G2 phases, but increased for the G0 phase. For 1 Gy irradiation, the increases of cells in all phases were delayed relative to the control. For 5 Gy irradiation, cells in all phases except G0 significantly decreased with time, and the number of G0 cells remained extremely low throughout the simulation period. The number of G1 arrested cells after 0.1–2 Gy irradiation slightly increased, compared with that for the control. Due to DSB repair, the cells arrested in the G1 phase by exposure to 0.1–2 Gy irradiation were released from the G1/S checkpoint and entered back into the cell cycle. Thus the number of G1 arrested cells decreased with time. For exposure to 5 Gy irradiation, the number of arrested cells increased greatly with different kinetics for each arrest mode: the rate of increase strongly correlated with the threshold values. Because almost all cells in the case of 5 Gy irradiation showed cell-cycle arrest and the number of G1 cells decreased, the number of G1 arrested cells was smaller than that in the case of 2 Gy irradiation. The time courses of population growth for each radiation dose are shown in Fig. 17. In area A, the population growth for 0.1–0.5 Gy exposure was similar to that of the control. For the higher dose range of 1–5 Gy, the rate of increase was suppressed because almost all cells were arrested by the intercellular signaling. From Fig. 17b, it can be seen that the number of cells decreased with dose but the decrease was not proportional to dose.

### Contribution of each intercellular signaling pathway

We now focus on areas B and C after irradiation (Figs. 13, 14, 15, 16 and 17). We scored cells with the exception of irradiated cells. In all cell-cycle phases, the numbers of cells in both areas decreased with time when exposed to 5 Gy (Figs. 13 and 14). In particular the number of cells strikingly decreased in the area B compared to area C due to the quantities of virtual signals in the two pathways. The cells in area B received virtual signals through both the MDP and the GJP, while the cells in area C received signals only through the MDP.

From Figs. 15 and 16, it can be seen that the total numbers of arrested cells for the control were negligible. For 0.5–5 Gy exposure, the number of G1 arrested cells in area B increased with time, and was similar to that in area C except for 5 Gy. The numbers of S, G2, and M arrested cells in area B for 1–5 Gy doses was smaller than those in area C: arrested cells were more efficiently accumulated in the population of area B larger than in the population of area C. From Fig. 17, it can be seen that the time course and the dose response of populations in area B were similar to those of populations in area C.

## Discussion and conclusions

**Fig. 18:**
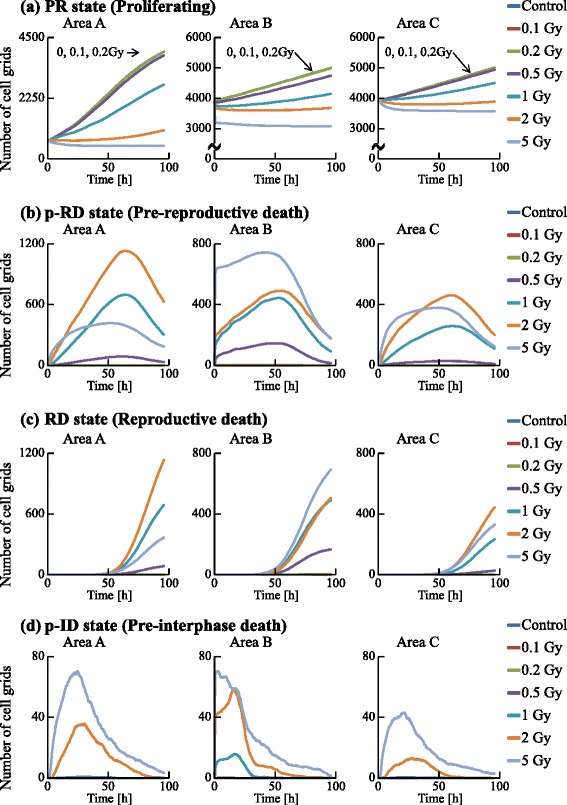
Time courses of the numbers of cells in the PR, p-RD, RD, and p-ID states. The numbers of cell grids in the PR state, the p-RD state, the RD state, and the p-ID state were counted at each time. The horizontal axis indicates the time. Each color represents a radiation dose

To investigate the dynamics of a cell population, the numbers of cells in the PR state, the pre-reproductive death (p-RD) state, the reproductive death (RD) state, and the pre-interphase death (p-ID) state were analyzed as shown in Fig. 18. The number of p-RD state cells reached a maximum 60–70 h after irradiation because the cells transited to the RD state after 24–72 h (1 to 3 cell divisions). The number of p-ID state cells reached a maximum 20–30 h after irradiation because the cells transited to interphase death within 1 cell division. The number of PR state cells decreased with dose because of cell-cycle arrest or cell death. During the 0–50 h period, the number of RD state cells was much smaller than the numbers of G1, S, and G2 arrested cells (Figs. 13 and 14). Thus, the cell-cycle arrest induced by bystander signals reduced the number of PR state cells soon after irradiation.

The number of arrested cells did not increase linearly with dose. This is due to the DSB thresholds determining cell-cycle arrest. Since we set the threshold at the G1/S checkpoint to be smaller than that at other checkpoints, G1 arrested cells accumulated in the population more than other arrested cells. Although the threshold at the G2/M checkpoint was set to half of that at the S/G2 checkpoint, the number of G2 arrested cells was more than twice the number of S arrested cells. Thus, the number of arrested cells depends sensitively on these thresholds.

To validate the simulation results, the dynamics of the model should be further examined by comparison with applicable experimental data. Although there have been few reports on bystander cell-cycle effects so far because of technical difficulties in tracking the cell cycle of individual cells, recent live cell imaging studies using fluorescent ubiquitination-based cell-cycle indicators (FUCCI) have enabled visualization of the cell cycle of individual cells [45]. Such an approach is expected soon to provide applicable data that can be used to refine and improve our model.

Using FUCCI cells, we recently determined cell-cycle periods after exposure of specific cells in a population to X-rays [17] or an X-ray microbeam [46]. To validate the present simulation results, particularly Figs. 13, 14, 15 and 16, we have also been examining whether non-irradiated cells undergo cell cycles in a colony in which some cells were exposed to an X-ray microbeam (to be submitted). Thus, the present model could be used to predict dynamic changes from cycle-progressing cells to arrested cells, or the growth of cellular populations before performing irradiation experiments.

The GJP contributes more to bystander effects than the MDP, as shown in Figs. 13, 14, 15 and 16. For 5 Gy irradiation, the average number of DSBs in area B reached a maximum at 1 h after irradiation, and the maximum was 47 DSBs/cell in the G1 phase. In area C, the average value reached a maximum at 10 h, and the maximum was 17 DSBs/cell in the G1 phase. These results clearly show that the GJP contributed significantly to the induction of DSBs. Furthermore, the arrested cells were more efficiently accumulated in the population of area B than in the population of area C, indicating that signaling through the GJP plays a significant role in bystander cell-cycle effects.

Experimentally suppressing all MDP or GJP signals would highlight the particular contribution of a specific pathway; however, it would be technically difficult to completely quench the signals using high concentrations of scavenging chemicals, which might cause undesirable side-effects in the cells. The present model is therefore a powerful tool for predicting bystander effects from the actions of intercellular signaling pathways.

## Additional file

Additional file 1
**This file contains the method for discretization of the diffusion equation and Figure.** Modeling of the intercellular signaling was based on the discretized diffusion equation. (PDF 65.9 kb)

## Abbreviations

DSB: Double-strand break
GJP: Gap junctional pathway (intercellular signaling pathway)
MDP: Medium-mediated pathway (intercellular signaling pathway)
