# Supplementary material for: Cellular automaton-based model for radiation-induced bystander effects
Source: BMC Syst Biol. 2015 Dec 7;9:90. doi: 10.1186/s12918-015-0235-2 (PMC4672575; doi:10.1186/s12918-015-0235-2)
Supplement: Additional file 1 — This file contains the method for discretization of the diffusion equation and Figure. Modeling of the intercellular signaling was based on the discretized diffusion equation. (PDF 65.9 kb) [file 12918_2015_235_MOESM1_ESM.pdf]

Additional File 1 for Cellular automaton-based model for radiation-induced bystander effects: **Discretization of the diffusion equation**

Yuya Hattori, Akinari Yokoya, Ritsuko Watanabe

Japan Atomic Energy Agency, 2-4, Shirakata Shirane, Tokai, Ibaraki, Japan

November 6, 2015

Discretization of the diffusion equation

Intercellular signaling in our model is based on the discretized diffusion equation. The diffusion equation in two dimensions can be written as

$$\frac{\partial \phi(x,y,t)}{\partial t} = {}^{\phi}w(\frac{\partial^2 \phi(x,y,t)}{\partial x^2} + \frac{\partial^2 \phi(x,y,t)}{\partial y^2}) \qquad (1)$$

where $\phi(x,y,t)$ is the density of the diffusing material at location (x,y) and ${}^{\mathrm{X}}w$ is the diffusion coefficient. Using the forward finite difference approximation for time, $\partial\phi(x,y,t)/\partial t$ is discretized as follows:

$$\frac{\partial \phi(x,y,t)}{\partial t} \cong \frac{\phi(x,y,t+\Delta t) - \phi(x,y,t)}{\Delta t} \qquad (2)$$

where Δt is a time interval. Using the central difference approximation for space at time t, $\partial^2\phi(x,y,t)/\partial x^2$ is discretized as follows:

$$\frac{\partial^2 \phi(x,y,t)}{\partial x^2} \cong \frac{\phi(x+\Delta x,y,t) - 2\phi(x,y,t) + \phi(x-\Delta x,y,t)}{\Delta x^2} \qquad (3)$$

where Δx is a spatial interval for the x axis. These discretizations are called the forward-time central-space (FTCS) method. Based on the FTCS method, the diffusion equation can be discretized as follows:

$$\begin{aligned}\frac{\phi(x,y,t+\Delta t) - \phi(x,y,t)}{\Delta t} &= {}^{\phi}w(\frac{\phi(x+\Delta x,y,t) - 2\phi(x,y,t) + \phi(x-\Delta x,y,t)}{\Delta x^2} \\ &+ \frac{\phi(x,y+\Delta y,t) - 2\phi(x,y,t) + \phi(x,y-\Delta y,t)}{\Delta y^2}) \quad (4)\end{aligned}$$

where Δy is a spatial interval for the y axis. In a cellular automaton, location is represented by a grid position such as (i,j) (Figure 1a). We assumed that the density, $\phi(x,y,t)$, in the grid square (i,j) is uniform and is represented by $\phi_{i,j}(t)$. In our model, $\Delta x = \Delta y = d$. Therefore, equation (4) becomes

$$\begin{aligned}\phi_{i,j}(t+\Delta t) = \phi_{i,j}(t) &+ \frac{\Delta t}{d^2}{}^{\phi}w(\phi_{i+1,j}(t) - 2\phi_{i,j}(t) + \phi_{i-1,j}(t) \\ &+ \phi_{i,j+1}(t) - 2\phi_{i,j}(t) + \phi_{i,j-1}(t)) \qquad (5)\end{aligned}$$

Representing the positions of the four nearest neighbors surrounding grid square (i,j) by (k_1,l_1), equation (5) becomes

$$\phi_{i,j}(t+\Delta t) = \phi_{i,j}(t) \quad + \quad \frac{\Delta t}{d^2}{}^{\phi}w(\sum_{k_1,l_1}\phi_{k_1,l_1}(t) - \phi_{i,j}(t)) \tag{6}$$

where (k_1, l_1) runs over $(i+1, j)$, $(i-1, j)$, $(i, j+1)$, and $(i, j-1)$. This equation shows that an intercellular signal in (i, j) diffuses into the four neighbors during Δt. We also considered intercellular signaling for the skew grid squares, $(i+1, j+1)$, $(i-1, j+1)$, $(i+1, j+1)$, and $(i-1, j-1)$ during Δt (Figure 1b). Based on the FTCS method, the diffusion equation could be discretized using skew location as follows:

$$\begin{aligned}\frac{\phi(x,y,t{+}\Delta t) - \phi(x,y,t)}{\Delta t} \quad &= {}^{\phi}w(\frac{\phi(x{+}\Delta x, y{+}\Delta y, t) - 2\phi(x,y,t) + \phi(x{-}\Delta x, y{-}\Delta y, t)}{\Delta x^2 + \Delta y^2} \\ &+ \frac{\phi(x{-}\Delta x, y{+}\Delta y, t) - 2\phi(x,y,t) + \phi(x{+}\Delta x, y{-}\Delta y, t)}{\Delta x^2 + \Delta y^2}\end{aligned} \tag{7}$$

Using grid square position and spatial interval, d, equation (7) becomes

$$\phi_{i,j}(t+\Delta t) = \phi_{i,j}(t) \quad + \quad \frac{\Delta t}{2d^2}{}^{\phi}w(\sum_{k_2,l_2}\phi_{k_2,l_2}(t) - \phi_{i,j}(t)) \tag{8}$$

where (k_2, l_2) runs over $(i+1, j+1)$, $(i-1, j+1)$, $(i+1, j+1)$, and $(i-1, j-1)$. Adding equation (6) to (8), we get the following discretized diffusion equation.

$$\begin{aligned}\phi_{i,j}(t+\Delta t) = \phi_{i,j}(t) \quad &+ \quad \frac{\Delta t}{4d^2}{}^{\phi}w[2\sum_{k_1,l_1}(\phi_{k_1,l_1}(t) - \phi_{i,j}(t)) \\ &+ \quad \sum_{k_2,l_2}(\phi_{k_2,l_2}(t) - \phi_{i,j}(t))].\end{aligned} \tag{9}$$

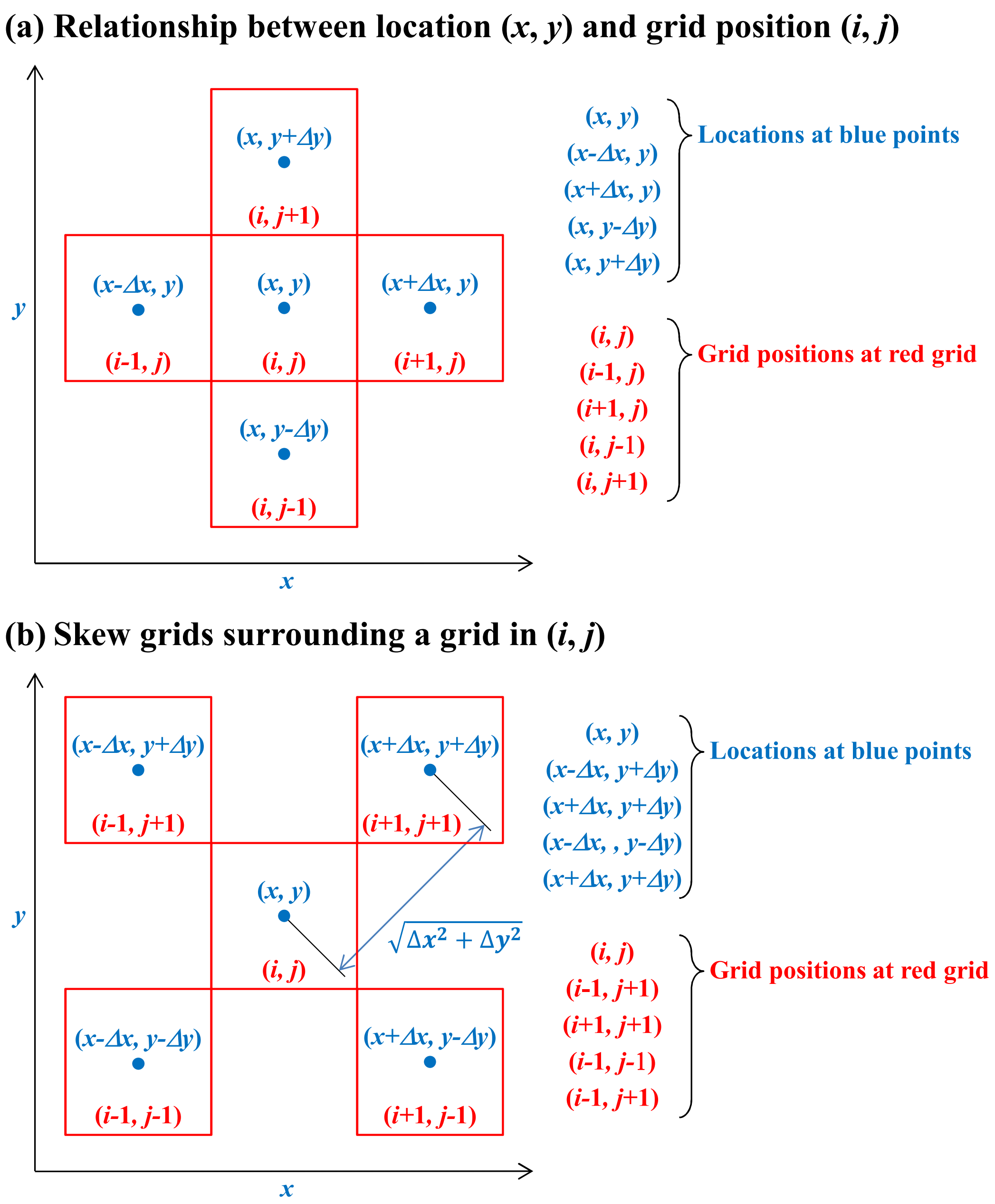

Figure 1: Schematic layout of a grid square at (i, j) and its surroundings. Blue points and red lines represent the location and the grid indices, respectively. (a) The positions of nearest neighbors of grid square (i, j). (b) The positions of skew nearest neighbors surrounding grid square (i, j).
